# A neurological approach to biopsychosocial medicine: Lessons from irritable bowel syndrome

**DOI:** 10.1186/1751-0759-5-1

**Published:** 2011-01-31

**Authors:** Shin Fukudo

**Affiliations:** 1 Department of Behavioral Medicine, Tohoku University Graduate School of Medicine Sendai, Japan

## 

Modern people are still influenced by the mind-body dualism of Rene Descartes. Some of these people said "Anxiety aggravated the symptoms of these patients," a notion that most people probably consider natural. By contrast, I would like to criticize this concept because anxiety is posited as an independent driving force that actively changes one's body. However, anxiety per se is a subjective feeling produced by the brain and somatic signals from the body. Actually, neural activities in the central and autonomic nervous systems along with endocrine and immune function change the body. From this point of view, anxiety is not the CAUSE of mind-body changes but the RESULT of them. The origin and synthetic processing of emotion are more important than simple idea that the "mind" directly changes the body. Descartes' mind-body dualism conceals, in our view, how diseases should be recognized and analyzed.

Functional gastrointestinal disorders are useful for illustrating how the rationale of "nervism" of my notion is manifest. In particular, irritable bowel syndrome (IBS) is a prototype of functional gastrointestinal disorders [1], and the biopsychosocial model is well fit to IBS [1]. Although genes, inflammation, gut microbiota, psychosocial stress, and early learning may play an important role in the pathogenesis of IBS [2,3], brain-gut interactions cannot be excluded from the pathophysiology of IBS [4]. Brain imaging studies have clarified the roles of the anterior cingulate cortex, amygdala, insula and the brain stem in response to visceral stimulation [2,5,6]. These structures produce both visceral pain and negative emotion that are typical symptoms of IBS patients. Some researchers interpret the anxiety and depression of IBS patients as "noise" or "confounding factors" that require control. However, IBS patients naturally have negative emotion. Some IBS patients may not have anxiety or depression at the clinically diagnostic level, but a high percentage of IBS patients show increased levels of anxiety and depression. Therefore, the concept that "pure IBS" pathophysiology is completely separated from anxiety and depression is influenced by the Descartes' mind-body dualism and may hinder our ability to see the true nature of the mind-body relationship.

The clinical usefulness of psychological treatments for IBS patients is evidence of the rationale of the biopsychosocial model. The theoretical background of psychotherapies for IBS is as follows. First, IBS patients have deranged life styles characterized by more perceived stress, more irregular sleep and more irregular meals [7]. Reduction of these risk factors is the first step to minimize the exacerbating factors of IBS. Second, the doctor-patient relationship is important for effective treatment. A positive physician-patient interaction is associated with a good prognosis of IBS [8]. Third, the close relationship between daily stress or hassles and IBS symptoms is present [9]. The stress response should be managed. Fourth, IBS patients show high response to placebo [10]. This phenomenon means that suggestion and expectation greatly influence the therapeutic effect. Fifth, IBS patients have high levels of anxiety, depression, and somatization [11]. Not only neuropharmacotherapies but also psychotherapies are indispensable for these co-morbid disorders whose improvement often alleviates IBS symptoms. Sixth, intractable IBS patients have often experienced traumatic life events [12]. Improved brain function has been reported after treatment including psychotherapy [13]. Seventh, the effects of health beliefs and learned behaviors may adversely affect outcome.

There is evidence from a systematic review and meta-analysis of rigorously executed trials on IBS that psychological treatment in general is effective for IBS [14]. Hypnotherapy is a representative strategy for refractory IBS [15]. Autogenic training is auto-hypnosis with the goal of being able to self-administer suggestions of relaxation. We reported that autogenic training is effective for intractable IBS patients [16]. Cognitive-behavioral therapy (CBT) focuses on ways to increase or decrease a particular behavior that addresses automatic thoughts and irrational beliefs. CBT was found to be more effective than an educational intervention [17]. Psychological treatments are interpreted as how humans have been influenced by others. During psychological treatments, function of the specific brain regions in patients is altered. Good examples are the decreased activities of the anterior cingulate cortex during hypnotic modulation of pain [18] and the increased activities of the anterior cingulate cortex during empathy to pain [19]. Therefore, how neural processing of interoception is modified after the activation of specific brain regions, including the mirror neuron system, should be explored. Further research on neurological approaches to biopsychosocial medicine will be valuable.

## Abbreviations

IBS: irritable bowel syndrome; CBT: cognitive-behavioral therapy

## Competing interests

The authors declare that they have no competing interests.
